# Dynamics of fluid displacement in mixed-wet porous media

**DOI:** 10.1098/rspa.2020.0040

**Published:** 2020-08-05

**Authors:** Alessio Scanziani, Qingyang Lin, Abdulla Alhosani, Martin J. Blunt, Branko Bijeljic

**Affiliations:** Department of Earth Science and Engineering, Imperial College London, SW7 2AZ London, UK

**Keywords:** wettability, multiphase flow, mixed-wet, contact angle, X-ray imaging, porous media

## Abstract

We identify a distinct two-phase flow invasion pattern in a mixed-wet porous medium. Time-resolved high-resolution synchrotron X-ray imaging is used to study the invasion of water through a small rock sample filled with oil, characterized by a wide non-uniform distribution of local contact angles both above and below 90^°^. The water advances in a connected front, but throats are not invaded in decreasing order of size, as predicted by invasion percolation theory for uniformly hydrophobic systems. Instead, we observe pinning of the three-phase contact between the fluids and the solid, manifested as contact angle hysteresis, which prevents snap-off and interface retraction. In the absence of viscous dissipation, we use an energy balance to find an effective, thermodynamic, contact angle for displacement and show that this angle increases during the displacement. Displacement occurs when the local contact angles overcome the advancing contact angles at a pinned interface: it is wettability which controls the filling sequence. The product of the principal interfacial curvatures, the Gaussian curvature, is negative, implying well-connected phases which is consistent with pinning at the contact line while providing a topological explanation for the high displacement efficiencies in mixed-wet media.

## 1. Introduction

If the angle that the interface between two fluids forms with a solid surface (the contact angle), is both lower and higher than 90^°^, meaning that there is a mix of hydrophobic and hydrophilic regions, a material is defined as being mixed-wet [1]. Flow in mixed-wet porous media is omnipresent in nature [2]. For example, lotus and rice leaves [3,4], butterfly wings and gecko feet [5,6], and also human skin [7] are natural porous systems which are not wetted by water and show different grades of mixed wettability (or hydrophobicity). Mixed wettability is studied for several applications: in the fabric industry, the wettability of textiles is altered to form anti-fogging, self-cleaning materials [8]; in the medical and cosmetic sectors, wettability controls skin friction and lubrication [9], while in earth science the wettability of the subsurface governs secure storage of CO_2_ [10,11], as well as oil recovery [12,13].

To investigate two-phase flow invasion patterns in a mixed-wet porous medium, we will use high-resolution X-ray microtomography which provides non-destructive, three-dimensional (3D) visualization of the fluids inside different porous materials, from rocks [14,15] to termite nests [16]. This technology has significantly increased the understanding of flow in porous media, including wettability characterization with direct measurement of *in situ* contact angles [1,17].

Studies of mixed-wet porous rocks using laboratory-based microtomography show that the wettability has an impact on secure trapping of CO_2_ [18] and for oil recovery for which mixed-wet conditions are favourable [19]. While these studies give valuable insights on the end states, they do not capture the displacement or pore-filling sequence, as several minutes (or hours) are required to obtain a single scan. The high photon flux available at synchrotron radiation sources, instead, allows for fast imaging and it can be used to study the dynamics of fluid invasion in porous media [20,21].

The physics of invasion patterns has been studied in systems with uniform contact angles [22–25]. Drainage, where a non-wetting phase displaces the wetting phase, is an invasion percolation process where fluid advances in a connected front from pore to pore through the restrictions, or throats, between pores [24,25]. Filling a wide region of the pore space requires a lower capillary pressure, *P*_*c*_. The Young–Laplace equation defines the capillary pressure which, for throats with a cylindrical cross-section of radius *r*, gives

1.1 $$P_{c}=\frac{2\sigma \cos\theta }{r},$$

where *σ* is the interfacial tension between the two fluids and *θ* is their contact angle [2].

Synchrotron and confocal imaging studies have directly observed the dynamics of drainage at the pore scale, showing that this process is characterized by fast invasions of multiple pores (Haines jumps) [26,27], which cause interface recession and distal snap-off in other pores [28]. Snap-off is the filling of throats by the wetting phase, which can disconnect the non-wetting phase [29,30].

If we decrease the contact angle, and the invading phase becomes more wetting, there is a transition from invasion percolation towards frontal advance with displacement controlled by cooperative pore filling in the absence of wetting layer flow—the Cieplak–Robbins transition [31–33].

However, in most natural systems, the wetting phase can also flow in corners and roughness in the pore space (layer flow) [34]. In this case, the displacement of the non-wetting phase by a wetting phase (imbibition) has a more complex dynamics with snap-off [24,35–37]. The displacement becomes percolation-like as the wetting phase fills the narrowest pores and throats throughout the domain [2,34].

A recent study of displacement patterns, in micromodels with a uniform contact angle, identified this transition from invasion percolation to connected advance, with layer flow for the most wetting systems. Varying the capillary number and the contact angle of the system, it was shown that the 3D structure has a strong impact on multiphase flow even in quasi-two-dimensional (2D) patterned microfluidic flow cells and that layer flow affected the dynamics [38].

However, the displacement dynamics have not been studied in a mixed-wet system with 3D imaging techniques. In particular, we study displacement patterns in a porous rock with a non-uniform distribution of contact angles both above and below 90^°^. We dynamically image capillary-dominated waterflooding, after primary drainage, at high spatial and temporal resolution, using a synchrotron X-ray source. Our objective is to understand pore-scale displacement physics in mixed-wet media using observations and analysis of displacement patterns, and several multiphase flow descriptors including contact angle, interfacial curvatures, capillary pressure and interfacial area measured on the X-ray images.

From the experiments, we observe a distinct type of displacement pattern, dissimilar to that seen either in drainage or imbibition: there is advance of a connected water front, through the centre of the pores, without evidence of layer flow. The filling sequence is not in order of pore size, while contact angle hysteresis does not allow for interface retraction and snap-off.

Pinning and depinning of the contact line had been previously related to the thermodynamics of wetting of 2D surfaces [39,40]. We show that, inside the 3D mixed-wet medium studied, the filling order is controlled by the thermodynamic contact angle [41], which represents the energetic threshold to be overcome for depinning of the interface for displacement.

Local pinning of the contact line also drives the interfaces to arrange as minimal surfaces, with the principal curvatures of opposite signs in orthogonal directions [42]. The consequent negative Gaussian curvature indicates well-connected phases, favouring high displacement efficiency [22].

Our findings can be extended to other kinds of porous materials. They have numerous practical applications such as evaporation of liquids inside hydrophobic materials [43], spray cooling, nanoassembly and DNA stretching, for instance, where a mixed-wet state could be designed for optimal process performance.

## 2. Material and methods

### (a) Materials

We used a small cylindrical sample of Ketton limestone, 16.1 mm long and with a diameter of 5.8 mm. This was drilled from a larger piece of rock, where the permeability was measured to be 2.8 × 10^−12^ m^2^ [44]. The Helium porosity of the sample was 28%, and it is chemically composed of greater than 99% calcite [35]. The experimental fluids were doped to increase their X-ray attenuation values, to enhance the image contrast [45] by using 15% by weight iododecane in *n*-decane for the oil phase and 20% by weight potassium iodide (KI) in water, which we call the water phase. Table 1 provides the physical properties of the rock and fluids.

**Table 1.** Physical properties of the fluids and the rock used in the experiment. Density was measured at 40^°^ and 7.6 MPa. The viscosity of water was measured at 50^°^ and 10 MPa from [46] and of decane at ambient condition from [47]. The interfacial tension was measured directly using the pendant drop method [48,49]. The total porosity was measured using a Helium porosimeter, while the macro porosity was computed from the images, considering only the pore space which could be resolved with the given resolution (3.5 μm). The pore volume, PV, was computed considering the macro porosity. For the chemical composition refer to [28].

fluid	density (kg m^−3^)	viscosity (μ Pa s)	interfacial tension (mN m^−1^)
deionized water + 20%w KI	1154	547	*σ*_ow_ = 52.1 ± 5
*n*-decane + 15%w C_10_H_21_I	715	1088	

rock	porosity	chemical composition	dimensions
Ketton	total 28%	>99% calcite	*d* = 5.8 mm, *L* = 16.1 mm
	macro pores 14.3%		PV = 6.03 × 10^−8^ m^3^

### (b) Establishing wettability

The wettability of the rock was altered prior to the experiment. The sample was first cleaned using methanol and dried in an oven for 24 h, to remove any impurities. Then, the pore space was fully saturated with formation brine, replicating the chemical composition of brine in a giant producing reservoir in the Middle East while the pressure and temperature were raised to reservoir conditions (80^°^C and 10 MPa). Crude oil, extracted from the same reservoir, was injected, with increasing flow rate starting at 0.01 ml min^−1^, up to 0.1 ml min^−1^, for a total of 40 pore volumes (PV), from the bottom of the sample. The flow was then reversed and fresh oil was injected from the top of the sample, with the same flow rates and total volume. During the first week, 5 PV of oil were injected each day, at a flow rate of 0.05 ml min^−1^. After four weeks, the sample was removed from the core holder and left in a closed crude oil bath at 80^°^C for three more months. This process alters the wettability of the rock from being water–wet, where, as discussed in the introduction, waterflooding is an imbibition process, to a mixed-wet state with a distinct displacement dynamics which we analyse in this paper.

### (c) Experiment and imaging

The sample was transferred to the I13-2 beamline of the Diamond Light Source synchrotron facility (Harwell Campus, Didcot, Oxfordshire) in the crude oil bath, at ambient temperature. It was then mounted in a core holder and the crude oil was replaced with the oil phase (doped decane), to avoid the formation of an emulsion during the experiment [50], by flushing 20 PV of oil at a flow rate of 0.1 ml min^−1^. We waited for 2 h for the fluids to reach equilibrium. Temperature and pressure were then increased to the experimental conditions (50^°^C and 8 MPa) and we started to inject water at low flow rate (0.50 μl min^−1^), corresponding to a capillary number of *Ca* = *μq*/*σ* of 3.3 × 10^−9^, where μ is the injected water viscosity, *q* is the Darcy flux (volume injected per unit area per unit time) and *σ* is the interfacial tension (table 1 shows the physical properties of rock and fluids).

Sixty-five tomograms were acquired during water injection, every 74 s, for a total of 4706 s (78.4 min), with an exposure time of 0.06 s and using 900 projections. The time required for each scan includes 54 s of image acquisition and 20 s of file transfer and back-rotation of the sample. Before and after water injection, static high-quality images were taken using a higher number of projections (3000) and exposure time (0.08 s). Each image contains 1280 × 1284 × 1280 voxels with a side length of 3.5 μm.

### (d) Image processing and segmentation

**Figure 1.**
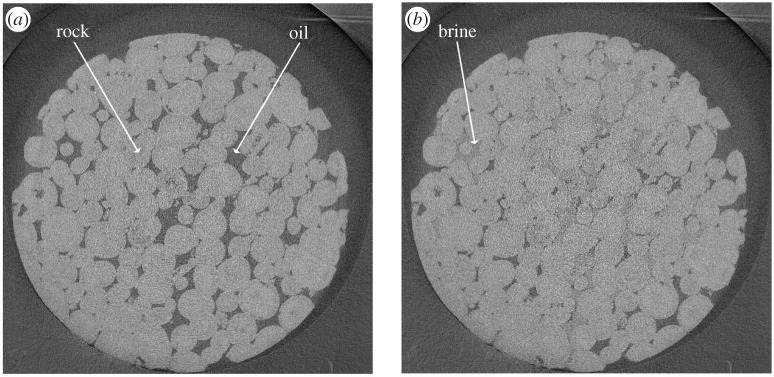
2D slices of the 3D tomograms as they appear after reconstruction. (*a*) A slice of the image before water injection, while the image in (*b*) was taken at the end of water injection.

**Figure 2.**
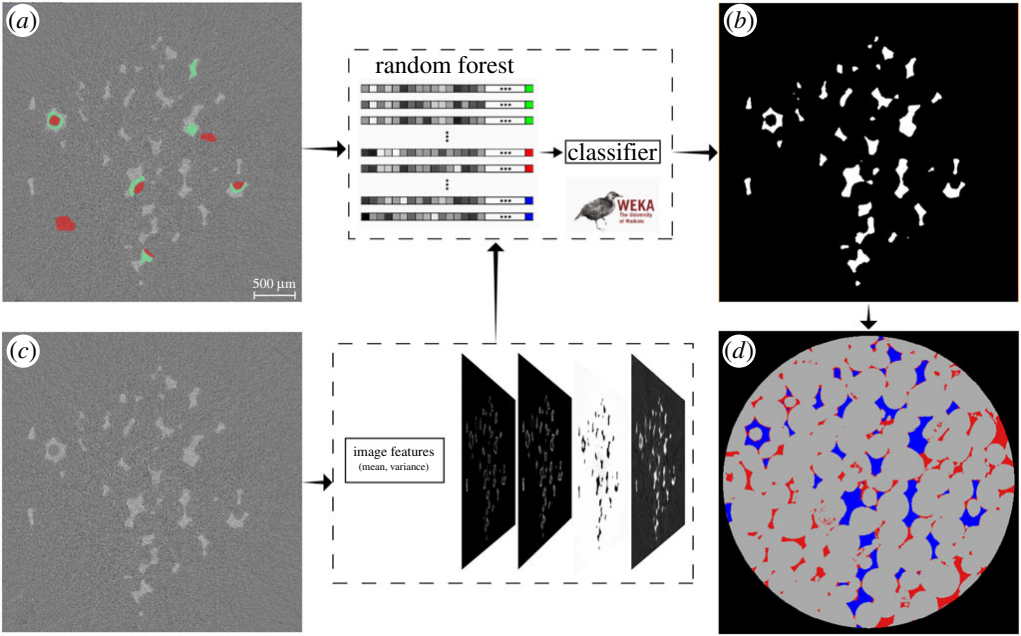
Workflow for image segmentation. The images before water injection (figure 1*a*) were subtracted from the images with water (figure 1*b*). The resulting differential image is shown in (*c*) of this image: brighter areas correspond to the places invaded by water. We created a training dataset with manual classification of invaded voxels (green in *a*) versus non invaded by water (red). The machine learning WEKA algorithm computes image features (mean and variance) from the differential image, and these are given as input, together with the training dataset, to the random forest algorithm. The result is a classifier. We applied this classifier to the differential images obtained at each time step and obtained (*b*) binary images with 1 (white) where water invaded the sample and 0 (black) elsewhere. Combining these with the dry segmented image of the rock, we obtained the final three-phase images (*d*). In (*d*), grey is rock, red is oil and blue is water. (Online version in colour.)

The tomograms were reconstructed, obtaining 3D grey scale images of the rock and the fluids within it during the injection (figure 1). Image segmentation—the classification of each image voxels into either water, oil or rock—was performed using a machine learning method, called WEKA [51], which provides high-quality results, if correctly trained [52] (figure 2). We subtracted the images before water invasion (figure 1*a*) from those with water in the pores (figure 1*b*) to create a differential image. The result, shown in figure 2*c*, is an image where the voxels invaded by water can be clearly distinguished. The training dataset was created with manual labelling of the differential image (figure 2*a*). This was provided as an input, together with the differential images, to the WEKA segmentation random forest algorithm. The resulting binary images (figure 2*b*) were combined with the segmented image of the rock to obtain the final segmented images in which voxels of oil, water and rock were assigned to three discrete values (figure 2*d*).

To assess the accuracy of this segmentation method, we compared its results against those obtained with the more established watershed segmentation technique [53], applied to the static high-quality images obtained at the end of the injection, used as a benchmark. The two segmentation methods compared well, confirming previous work [52]. The watershed segmentation algorithm could not be applied to the dynamic images, due to the higher level of noise caused by fewer projections (Section 2c).

### (e) Throat occupancy and Pearson correlation coefficient

We used the maximal ball extraction code of a generalized network model [54] on segmented dry images of the porous medium. We determined occupancy as the phase residing in the centre of the throats, local restrictions of the pore space. The detailed procedure to obtain throat occupancy is described elsewhere [45] and is based on relating any point in the pore space to a maximal ball, which is the largest sphere that fits in the pore space at that point. The throat radius is the radius of the maximal ball that lies in the centre of a restriction in the pore space. We also studied the correlation between throats radius and the time at which they were filled, using the Pearson correlation coefficient *p* between two variables *x* and *y*

2.1 $$p=\frac{\sum_{i=1}^{n}(x_{i}-\bar{x})(y_{i}-\bar{y})}{\sqrt{\sum_{i=1}^{n}{\left(x_{i}-\bar{x}\right)}^{2}}\sqrt{\sum_{i=1}^{n}{\left(y_{i}-\bar{y}\right)}^{2}}},$$

where $\bar{x}$ and $\bar{y}$ are the estimated mean values, and *x*_*i*_ and *y*_*i*_ are single observations of the two variables. *r* can range between −1 and +1 representing perfect negative and positive correlation, respectively.

### (f) Pore scale descriptors, contact angle and capillary pressure

The segmented images, obtained as described in §2d, allow the computation of a large number of pore-scale properties, which quantify the physics of displacement in mixed-wet media. Saturations were computed using the pore space which can be resolved with the voxel size of 3.5 μm. We used previously developed algorithms to find the geometric contact angle [55], interfacial curvature, specific interfacial area, capillary pressure [42] and the thermodynamic contact angle [41].

## 3. Results and discussion

We start with (a) experimental observation of invasion patterns, and (b) analysis of the order of throat invasion. Then, (c) the geometric contact angle was computed to define the wettability in comparison with previous work, followed by (d) saturation, interfacial area, curvature and capillary pressure measurements. Next, (e) the Gaussian curvature was estimated to assess connectivity and finally, (f) the thermodynamic contact angle was computed using an energy balance [41].

### (a) Experimental observation of invasion patterns

**Figure 3.**
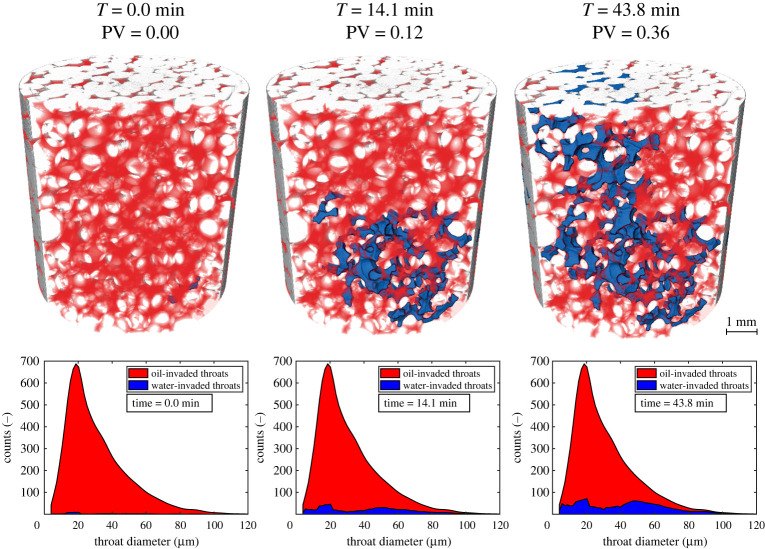
Top row: 3D rendering of rock (grey), oil (semi-transparent red) and water (blue) during waterflooding in the mixed-wet rock, at four time steps. Bottom row: Relation between the dimension of the throats and the phase occupying their centre at four times, during water invasion. Throats of a wide range of radii are invaded over time, showing that throat size is not the main parameter controlling filling. We started to count time since the onset of water invasion in the imaged domain. Pore volumes (PV) were computed considering the total volume of macro pores of the whole sample. (Online version in colour.)

To observe invasion patterns, we acquired images containing 1280 × 1284 × 1080 voxels with a side length of 3.5 μm every 74 s over a period of 78.4 min, giving 65 3D images in total, while water was injected at a flow rate of 0.5 μl min^−1^. This corresponds to a capillary number *Ca* = *μq*/*σ* of 3.3×10^−9^ (Section 2c). The top row of figure 3 shows that it took 28.3 min for the water front to reach the other side of the imaged domain, which accounted for about one-third of the total rock volume. Until breakthrough, 0.24 PV were injected. PV are computed considering the total macro pores of the whole sample which could be resolved with the image resolution of 3.5 μm (porosity and dimensions of the sample are available in table 1).

Firstly, we note that waterflooding in this mixed-wet rock happened differently to imbibition in water–wet systems: pore centres were invaded first, and oil was not isolated by snap-off [11,35].

Secondly, water displaced oil advancing as a connected phase, and once the pores were occupied, after breakthrough, water kept flowing in the same connected path, without further advancing and receding of the interfaces, due to interface pinning. This differs from previous visualizations of drainage, where distal snap-off and oil-filling events were observed [26,28,50].

Thirdly, figure 3 shows that the order in which pores were invaded differs from what had been previously observed during either imbibition or drainage, as throats with a wide range of size were invaded throughout the displacement: there was no filling preference based on throat radius, as further discussed in §3b.

**Figure 4.**
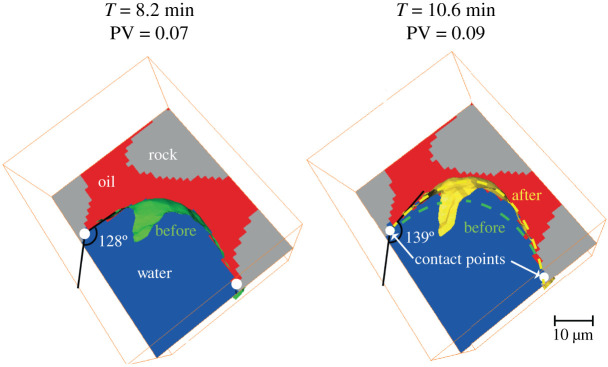
Visualization of a detail of the pore space where we can observe the pinning of the oil–water interface. During water injection, while the three-phase contact points (white dots) did not move, the interface changed its shape (from green to yellow), with a consequent increase in contact angle. Pore volumes (PV) were computed considering the total volume of macro pores of the whole sample. (Online version in colour.)

Finally, local pinning of the oil–water interface, see figure 4, provided evidence of a large contact angle hysteresis. Unlike in drainage [26], this pinning prevented the recession of the injected phase after invasion of a wide region of the pore space.

After pore invasion, the contact line found an equilibrium position and the contact points (where oil and water are in contact with the solid, shown in white in figure 4) did not move. The reason for this is the adhesion of surface-active components of the crude oil to the solid combined with roughness which limits the movement of the contact line [1]. However, the pressure of the water increased as it was injected. This caused a change in curvature and interfacial area (described next) and an increase in the local contact angle. Once the contact angle overcomes a certain threshold value, the interface is unpinned and it moves further in the pore space [39,40]. The system was characterized by contact angle hysteresis. In the example of figure 4 contact angle increased by approximately 11^°^ without unpinning and interface movement.

### (b) Order of throat invasion

As mentioned before, the classic invasion-percolation theory requires throats to be filled in decreasing order of size, as larger throats require lower capillary pressure to be invaded (equation 1.1). Pore-network models have been grounded on this basis and are able to predict the displacement sequence in water–wet media [56]. However, the bottom row of figure 3 seems to contradict this hypothesis for this mixed-wet porous medium, as small throats were also invaded at early stages (e.g. T=14.1 min, PV=0.12).

**Figure 5.**
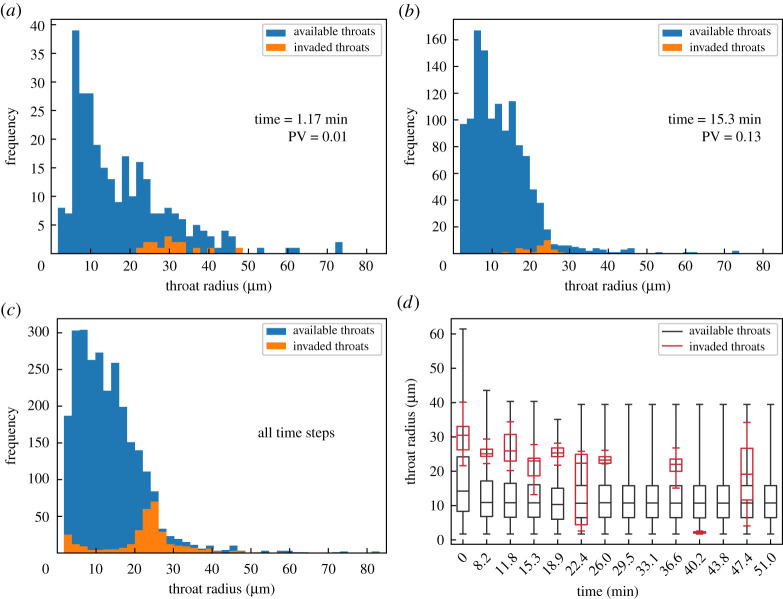
(*a*–*c*) At each time step, we identified the throats available for invasion (connected to the water front) and compared these with the ones which were actually invaded. (*d*) Box-plot of available and invaded throats at each time step. (Online version in colour.)

To investigate further this phenomenon, we considered the throats available to be filled at each time step *t* and compared their size with that of the throats which were actually filled by water at the following time step *t* + 1. Available throats are defined as throats which are connected to the water front at *t*. Figure 5 shows that, although there is a tendency for water to invade throats with a radius slightly higher than the average value (box-plots of figure 5*d*), a considerable number of throats with small radius were invaded, even when larger ones were available for invasion (figure 5*c*).

**Figure 6.**
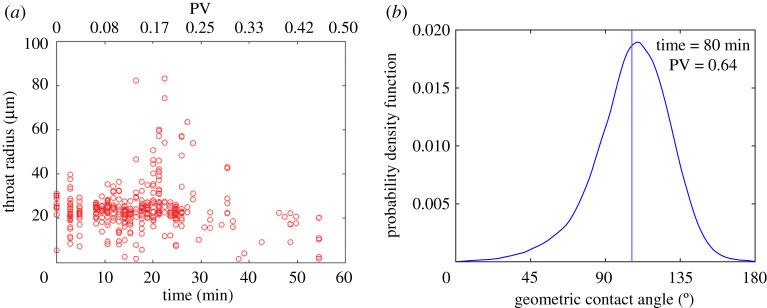
(*a*) Scatterplot where the radius of invaded throats is the dependent variable and the time at which they were filled is the dependent variable. (*b*) Geometric contact angle between water and oil on the high-quality image taken the end of waterflooding. The vertical line shows the average contact angle (109^°^). (Online version in colour.)

To quantify this observation, we used the Pearson correlation coefficient *p*, equation (2.1), and tested the correlation between the radius of the invaded throats and the time at which they were filled. Only throats available to be filled were considered at each time step. The Pearson correlation coefficient *p* (equation 2.1) was −0.03, which means that there is no correlation between throat radius and the order in which they are invaded, while invasion-percolation theory in a purely oil–wet medium would require a strong negative correlation. Figure 6*a* also shows that the data are scattered and no visual correlation can be noticed, confirming the quantitative result.

A number of pore-scale descriptors provide a complete physical and topological description of multiphase flow in porous media [11,57]. In the next sections, we use these to deepen our understanding of the observed invasion patterns, and ultimately to understand what is controlling flow in mixed-wet porous media.

### (c) Geometric contact angle

The *in situ* geometric contact angle, *θ*_*g*_, computed inside the pore space using 3D images at high resolution, can be used to define the wettability [2,17,55]. *θ*_*g*_ is usually defined at the end of waterflooding, imposing the constraint of constant curvature and computing the geometric angle between the oil–water interface and the solid [17,55]. We computed contact angles, using the method developed in [55] on the static images taken at the end of water injection (§2c). The resulting distribution is shown in figure 6*b*: the mean contact angle is 109^°^ and the standard deviation 23.2^°^. This indicates that the sample is indeed mixed-wet such that the solid does not display a strong preference for either water or oil, consistent with previous measurements on reservoir rocks [1].

### (d) Saturation, interfacial area, curvature and capillary pressure

**Figure 7.**
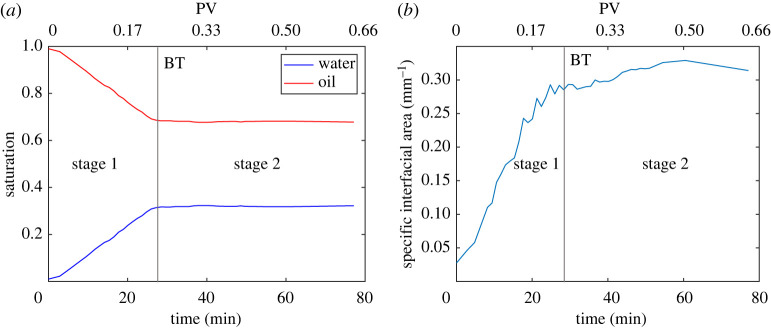
(*a*) Change in time of water and oil saturation, (*b*) specific interfacial area between oil and water. First and second stage are defined before and after breakthrough (BT), respectively. (Online version in colour.)

The saturation of water and oil was computed from the segmented images at each time step. Figure 7*a* shows that the water saturation linearly increased until breakthrough (28.3 min, or 0.24 PV, after the onset of water invasion in the imaged domain) and then it did not change further. The final oil saturation is related to the very low flow rate in the experiment, which was chosen to be able to follow water invasion pore by pore. The behaviour of saturation over time defines two stages of the injection: the first stage, from the beginning to breakthrough, with noticeable changes in volume fractions, and the second stage, where the injected water flows through the sample with little additional displacement, with pinned three-phase contact lines, as shown in figure 4. We also computed the saturation profile along the vertical direction, which did not show any significant variation. The pressure difference between the fluids due to the density difference is at most 69 Pa, which is much less than the capillary pressure, and so gravitational effects have a negligible influence on the saturation.

**Figure 8.**
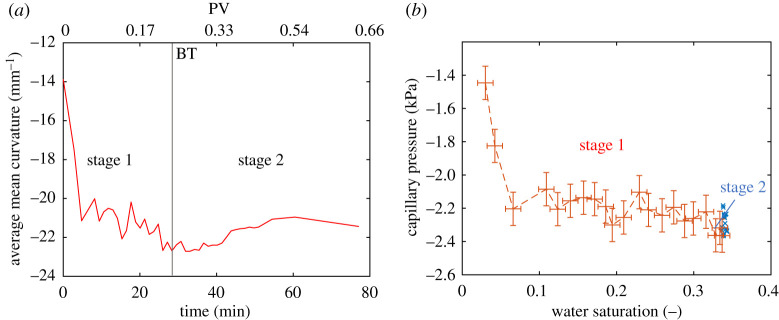
(*a*) Average mean curvature of the oil–water interface, (*b*) capillary pressure, before (dashed red line, 1st stage) and after (blue crosses, 2nd stage) breakthrough (BT). (Online version in colour.)

We extracted the oil–water interface which was smoothed to avoid voxelization artefacts [42]. A rigorous assessment of the accuracy of the smoothing and its impact on estimated areas and curvatures is provided elsewhere [58]: we can determine curvature to within 10% if the radius of curvature is 3 voxels or larger (more than 10 μm) or a curvature less than 100 mm^−1^, which, as we see later, is much larger than average values we measure. The resulting surfaces were used to compute the specific interfacial area between oil and water (interfacial area per unit volume), as a function of time. The principal curvatures *κ*_1_ and *κ*_2_ were also found: the mean curvature is defined as *κ* = (*κ*_1_ + *κ*_2_)/2. Figures 7*b* and 8*a* show that, while during the second stage volumes did not change, there was some relaxation of the fluid interfaces leading to an increase in curvature and interfacial area. The negative values of mean curvature show that on average it is water which bulges into oil, indicating slightly oil–wet or hydrophobic conditions on average, consistent with an average contact angle, figure 6, above 90^°^.

The capillary pressure was found from the Young–Laplace equation, *P*_*c*_ = 2*σκ*, using the measured interfacial tension, *σ* = 52.1 mN m^−1^ and plotted as a function of saturation in figure 8*b*. The values are slightly lower than those obtained in a sandstone sample whose contact angles were closer to 90^°^ [42]. The shape of the capillary pressure-saturation relationship is in line with the expected behaviour for a slightly oil–wet medium, starting from values approaching zero for low water saturation and plateauing afterwards, with a gradual decrease with saturation. After breakthrough (Stage 2 of water invasion, blue crosses), the capillary pressure increases slightly, due to local relaxation of the interfaces.

### (e) Gaussian curvature

**Figure 9.**
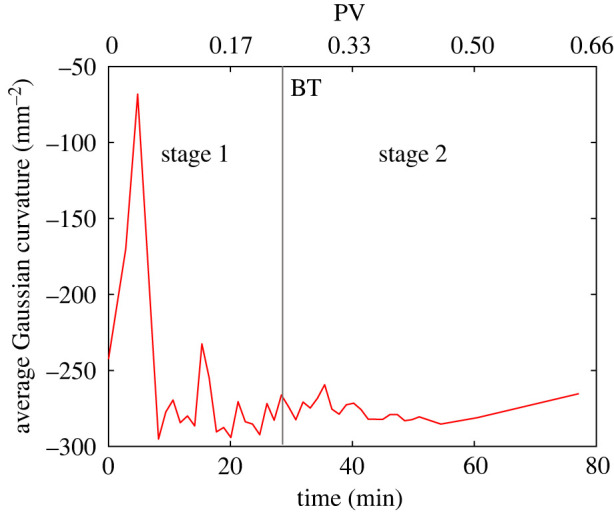
Gaussian curvature of the oil/water interface. Negative values imply high connectivity and favourable flow [57]. First and second stage are defined before and after breakthrough (BT), respectively. (Online version in colour.)

We computed the Gaussian curvature *G* = *κ*_1_ × *κ*_2_, shown in figure 9 as the average over the entire oil–water interface. Remarkably, we observe that the Gaussian curvature is negative: this means that in most cases the curvatures have opposite signs in orthogonal directions [42].

This observation of negative Gaussian curvature has some interesting consequences for flow. A negative value indicates a well-connected object [57] implying that oil and water can both flow, providing an explanation for the favourable recoveries seen in many mixed-wet systems [1,2,42]. Minimal surfaces are a special case with a mean curvature of zero: these form to minimize energy at pinned contacts in a range of circumstances from soap films, to foams and cell structures [42,59]. In our case, we indeed have pinned contacts, which drives the interfaces to be approximately minimal surfaces; however, displacement at a contact angle of greater than 90^°^ forces the curvature to be slightly negative to allow the water to displace oil in weakly oil–wet regions of the pore space.

### (f) Thermodynamic contact angle controls interface movement

The geometric contact angle *θ*_*g*_ shown in figure 6 characterizes the wettability of the system at rest after waterflooding. Since the fluid–fluid interfaces can hinge at fixed contact points, figure 4, *θ*_*g*_ does not necessarily indicate the contact angles associated with displacement, or those values that should be used in a pore-scale numerical model [60].

In contrast, the thermodynamic contact angle *θ*_*t*_ is computed from an energy balance, considering the changes in saturation and interfacial areas and assuming that there is little viscous dissipation caused by interface recession and rearrangement, which is valid for this experiment [41]:

3.1 $$\Delta a_{ws}\cos\theta_{t}=2\kappa \phi \Delta S_{w}+\Delta a_{wo},$$

where *ϕ* is the porosity (considering imaging resolved macro pores only), and Δ*S*_*w*_, Δ*a*_*wo*_ and Δ*a*_*ws*_ are the differences, between two subsequent time steps, in water saturation and in specific interfacial areas between water and oil, and water and solid.

In our experimental dataset *θ*_*t*_ can be computed only during the first stage of water invasion—before breakthrough—when both saturations and interfacial areas experience significant differences.

**Figure 10.**
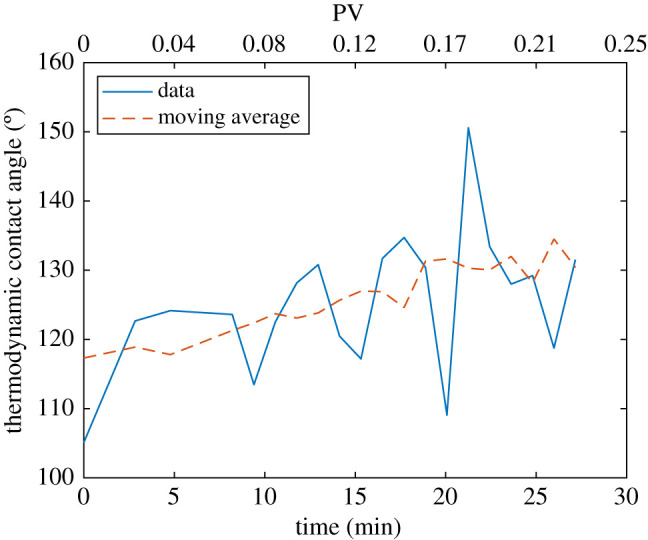
Thermodynamic contact angle, computed using equation (3.1) between two subsequent time steps. The 3-points moving average (dashed line) shows the increasing trend as the displacement proceeds. (Online version in colour.)

Figure 10 shows that *θ*_*t*_ has a value of approximately 110^°^ at the beginning of waterflooding. While water invades the pore space, an increasing trend is noticed, with a value close to 130^°^ at breakthrough. With respect to *θ*_*g*_, the values of *θ*_*t*_ are in the upper region of the probability distribution, higher than the mean geometric contact angle at the end of waterflooding. Previous work has shown that the agreement between simulation and experiment is improved by using contact angles in oil–wet pores that are higher than the geometric value [61].

During a displacement in a three-dimensional mixed-wet porous medium with a wide range of contact angle, it is the advancing contact angle which determines the threshold capillary pressure at which water can displace oil. Contact angle hysteresis causes this angle to be higher than *θ*_*g*_, which is measured at the end of waterflooding, when the fluids are at rest [39,40]. The reason for the large contact angle hysteresis is interface pinning, as described previously and shown in figure 4.

This contact angle hysteresis is why we did not observe interface recession and consequent distal and Roof snap-off events [28,50]. On the other hand, interface pinning explains why, while the invasion progresses, *θ*_*t*_ has to rise (figure 10). An increase in the local energy is required to overcome the interfacial forces caused by interface pinning, with a consequent increase of the thermodynamic contact angle as water invades more oil–wet pores.

This shows that, in mixed-wet systems, the dominant parameter which controls water invasion is the contact angle *θ*, and that the contact angle to be considered is *θ*_*t*_, as this encapsulates the energy required for the movement of the interface.

## 4. Conclusion

We have studied two-phase flow invasion patterns in a mixed-wet porous medium, using dynamic high-resolution X-ray synchrotron imaging. Water invades the pores through their centres, as a connected phase, without evidence of wetting layer flow. We identify a key signature of invasion patterns in three-dimensional mixed-wet media—a wide range of non-uniformly distributed local contact angles. We observe that the movement of water in the initially oil-filled medium is limited by interface pinning, responsible for contact angle hysteresis. This prevents interface recession and snap-off during the displacement. Water invasion does not happen in decreasing order of throat size, meaning that other parameters must control the filling sequence. The thermodynamic contact angle, which encapsulates an energy balance for pore invasion, increases until breakthrough, showing that it constrains pore filling in mixed-wet media. This new finding will be crucial for increasing the predictive abilities of pore-network models which simulate the flow in such mixed-wet porous media.

The presence of pinned interfaces drives the oil–water interfaces to become nearly minimal surfaces with a low mean curvature and a negative Gaussian curvature, meaning that the oil bulges into the water in one direction, while water bulges into oil in the other direction. This leads to well-connected phases and is the topological origin of high displacement efficiency in mixed-wet media [1,42].

We suggest that we could engineer a mixed-wet state in either natural or artificial materials to facilitate the simultaneous flow of two immiscible phases over a wide range of saturation. A control on the pinning of the fluid/fluid interface would allow the design of optimal conditions in porous materials, from oil recovery or CO_2_ storage in rocks, to favourable drying of fabrics, the manufacturing of nano devices and DNA stretching [43].

Future work can include the study of different porous media, to investigate the role of geometry and rock heterogeneity. Also, models such as Cassie–Wenzel wetting transition can be used to study the behaviour of contact angles including microstructural features such as surface roughness.

## Data accessibility

All the 3D images of the sample and the fluids within it during invasion have been uploaded to the Digital Rocks Portal (www.digitalrocksportal.org/projects/253) [62]. The scripts for image processing have been made open-access via GitHub (github.com/alessioscanziani).
